# Real world evaluation of kidney failure risk equations in predicting progression from chronic kidney disease to kidney failure in an Australian cohort

**DOI:** 10.1007/s40620-023-01680-2

**Published:** 2023-06-07

**Authors:** Sadia Jahan, Janine Hale, Eva Malacova, Cameron Hurst, Adrian Kark, Andrew Mallett

**Affiliations:** 1 Kidney Health Service, Royal Brisbane and Women’s Hospital, Herston, Brisbane, QLD 4029 Australia; 2 Central Northern Adelaide Renal and Transplantation Service, Royal Adelaide Hospital, Adelaide, SA 5000 Australia; 3 Renal Unit, Gold Coast University Hospital, Southport, QLD 4215 Australia; 4 QIMR Berghofer Medical Research Institute, Herston Road, Herston, Brisbane, QLD 4029 Australia; 5 Renal Unit, Mount Isa Base Hospital, Mount Isa, QLD 4825 Australia; 6 Department of Renal Medicine, Townsville University Hospital, Douglas, QLD 4814 Australia; 7 Institute for Molecular Bioscience and Faculty of Medicine, The University of Queensland, Brisbane, QLD 4072 Australia; 8 College of Medicine and Dentistry, James Cook University, Townsville, QLD 4814 Australia

**Keywords:** Progression prediction tool, Kidney failure risk equation KFRE, Predict progression, Kidney failure

## Abstract

**Background:**

Chronic kidney disease progression to kidney failure is diverse, and progression may be different according to genetic aspects and settings of care. We aimed to describe kidney failure risk equation prognostic accuracy in an Australian population.

**Methods:**

A retrospective cohort study was undertaken in a public hospital community-based chronic kidney disease service in Brisbane, Australia, which included a cohort of 406 adult patients with chronic kidney disease Stages 3–4 followed up over 5 years (1/1/13–1/1/18). Risk of progression to kidney failure at baseline using Kidney Failure Risk Equation models with three (eGFR/age/sex), four (add urinary-ACR) and eight variables (add serum-albumin/phosphate/bicarbonate/calcium) at 5 and 2 years were compared to actual patient outcomes.

**Results:**

Of 406 patients followed up over 5 years, 71 (17.5%) developed kidney failure, while 112 died before reaching kidney failure. The overall mean difference between observed and predicted risk was 0.51% (*p* = 0.659), 0.93% (*p* = 0.602), and − 0.03% (*p* = 0.967) for the three-, four- and eight-variable models, respectively. There was small improvement in the receiver operating characteristic-area under the curve from three-variable to four-variable models: 0.888 (95%CI = 0.819–0.957) versus 0.916 (95%CI = 0.847–0.985). The eight-variable model showed marginal receiver operating characteristic-area under the curve improvement: 0.916 (95%CI = 0.847–0.985) versus 0.922 (95%CI = 0.853–0.991). The results were similar in predicting 2 year risk of kidney failure.

**Conclusions:**

The kidney failure risk equation accurately predicted progression to kidney failure in an Australian chronic kidney disease population. Younger age, male sex, lower estimated glomerular filtration rate, higher albuminuria, diabetes mellitus, tobacco smoking and non-Caucasian ethnicity were associated with increased risk of kidney failure. Cause-specific cumulative incidence function for progression to kidney failure or death, stratified by chronic kidney disease stage, demonstrated differences within different chronic kidney disease stages, highlighting the interaction between comorbidity and outcome.

## Introduction

Chronic kidney disease (CKD) is a major public health issue [1, 2]. The rate of progression to kidney failure (KF) can vary widely [3, 4]. Some patients remain stable [4]; others progress rapidly requiring potential Kidney Replacement Therapy (KRT). Identifying those at risk of progression allows for early targeted interventions to slow kidney function loss and/or to prepare for KRT initiation [5, 6].

Tangri et al. published the Kidney Failure Risk Equation (KFRE) to predict risk of progression from CKD to KF, defined as the need to commence KRT, at 2 and 5 years [7]. Results are presented as a percentage risk and classified into three risk categories; low, intermediate and high. The group validated a three- (age, sex, eGFR[ml/min/1.73 m^2^]), four- (age, sex, eGFR, urine albumin-to-creatinine ratio (ACR)[mg/g]) and eight-variable KFRE (four-variables and serum bicarbonate, albumin, calcium and phosphate).

Initially developed and validated in two Canadian cohorts [7], the KFRE was subsequently validated in European [5], African-American [1], paediatric [8], and several multinational cohorts [1, 9]. Reporting of the application of KFRE in Australian cohorts, which we sought to do in our study, has been limited [10, 11].

## Methods

We included patients who were under the care of the Chronic Kidney Team, a multidisciplinary CKD programme provided by Royal Brisbane and Women’s Hospital (RBWH) within the Metro North Hospital and Health Service (MNHHS) on 1st January, 2013 (referral time range August 2001 to December 2012) who met KFRE calibration parameters [7] i.e., ≥ 18 but ≤ than 90 years of age with an estimated glomerular filtration rate (eGFR) < 60 mL/min per 1.73 m^2^ and not on KRT.

In their original paper [7], Tangri et al. described a total of seven models with an increasing number of variables starting with two (age and sex) through to a maximum of thirteen variables which included the above mentioned eight variables as well as diabetes mellitus, hypertension, systolic and diastolic blood pressure per 10 mmHg and body weight. Three models were validated [7] which we mirrored in our study: three variables (eGFR, age and sex), four variables (eGFR, age, sex and urinary ACR) and eight variables (eGFR, age, sex, urinary ACR, serum albumin; phosphate; bicarbonate and calcium).

Unique to this analysis, we undertook cause-specific cumulative incidence function for progression to KF or death, stratified by CKD stage.

As experienced by Tangri et al., measured urinary ACR was not available in most patients, and only available in 14% (*n* = 57) of our cohort [1]. For the remainder, urinary protein-to-creatinine ratios were transformed to the ACR using a previously reported equation [7].

### Model performance

The discrimination of the survival risk model developed using the Cox proportional hazards model was assessed using the concordance index (both Harrell’s C and Gӧnen and Heller’s K statistics). These concordance coefficients measure the discriminative power of prognostic models and they are equivalent to the receiver operating characteristic (ROC) curve for binary outcomes.

### Data analysis

The predicted and observed event probability estimates represent the mean predicted probability from the Cox regression model and the mean observed probability from the Kaplan Meier estimate divided into quintiles of predicted probability.

### Ethical approval

The RBWH Human Research Ethics Committee approved this retrospective cohort study as a low-risk quality assurance audit (HREC/18/QRBW/295) and waived individual patient consent.

### Patient and public involvement

Patients and the public were not involved in the undertaking of this research.

## Results

Four hundred seventeen patients were identified and followed up for 5 years. In 11 (2.6%) patients, at least one value required for the eight-variable KFRE was missing and so 406 patients remained for analysis (Table 1).

**Table 1:** Baseline characteristics

Characteristics	Total *N* = 406 (100%)	KF *N* = 71 (17.5%)	Death before event *N* = 112 (27.6%)	No event *N* = 223 (54.9%)
Age, mean (SD), years	70.9 (12.2)	64.1 (13.6)	78.0 (8.1)	69.6 (11.8)
Male sex	211 (52.0%)	44 (62.0%)	59 (52.7%)	108 (48.4%)
Caucasian ethnicity	373 (91.9%)	62 (87.3%)	108 (96.4%)	203 (91.0%)
Diabetes mellitus	219 (53.9%)	42 (59.2%)	71 (63.4%)	106 (47.5%)
History of current or previous smoking	205 (50.5%)	38 (53.5%)	54 (48.2%)	113 (50.7%)
Current smoking	34 (8.4%)	4 (5.6%)	6 (5.4)	24 (10.8)
eGFR (mL/min/1.73m^2^), mean (SD)	30.9 (11.7)	19.3 (9.1)	28.7 (9.6)	35.7 (10.4)
CKD stage 3	216 (53.2%)	8 (11.3%)	51 (45.5%)	157 (70.4%)
CKD stage 4	158 (38.9%)	41 (57.8%)	54 (48.2%)	63 (28.3%)
CKD stage 5	32 (7.9%)	22 (31.0%)	7 (6.3%)	3 (1.4%)
Serum calcium (mg/dL), mean (SD)	9.59 (0.47)	9.64 (0.56)	9.66 (0.55)	9.53 (0.39)
Serum phosphate (mg/dL), mean (SD)	3.7 (0.7)	4.4 (0.9)	3.6 (0.6)	3.5 (0.5)
Serum albumin (g/dL), mean (SD)	4.0 (0.4)	3.9 (0.3)	4.0 (0.4)	4.1 (0.3)
Serum bicarbonate (mEq/L), mean (SD)	25.1 (3.3)	24.0 (3.1)	24.6 (3.5)	25.7 (3.1)
Urine ACR (mg/g), median (IQR)	161.1 (470.8)	755.8 (1561.1)	185.9 (449.1)	92.9 (192.0)
Urine ACR < 30 (mg/g)	35 (8.6%)	0	7 (6.3%)	28 (12.6%)
Urine ACR 30–300 (mg/g)	237 (58.4%)	21 (29.6%)	64 (57.1%)	152 (68.2%)
Urine ACR > 300 (mg/g)	134 (33.0%)	50 (70.4%)	1 (36.6%)	43 (19.3%)

*IQR* interquartile range. To convert serum calcium to mmol/L, multiply by 0.25. To convert serum phosphate to mmol/L, multiply by 0.323. To convert serum albumin to g/L, multiply by 10. To convert urine ACR to mg/mmol, multiply by 0.113

**Fig. 1:**
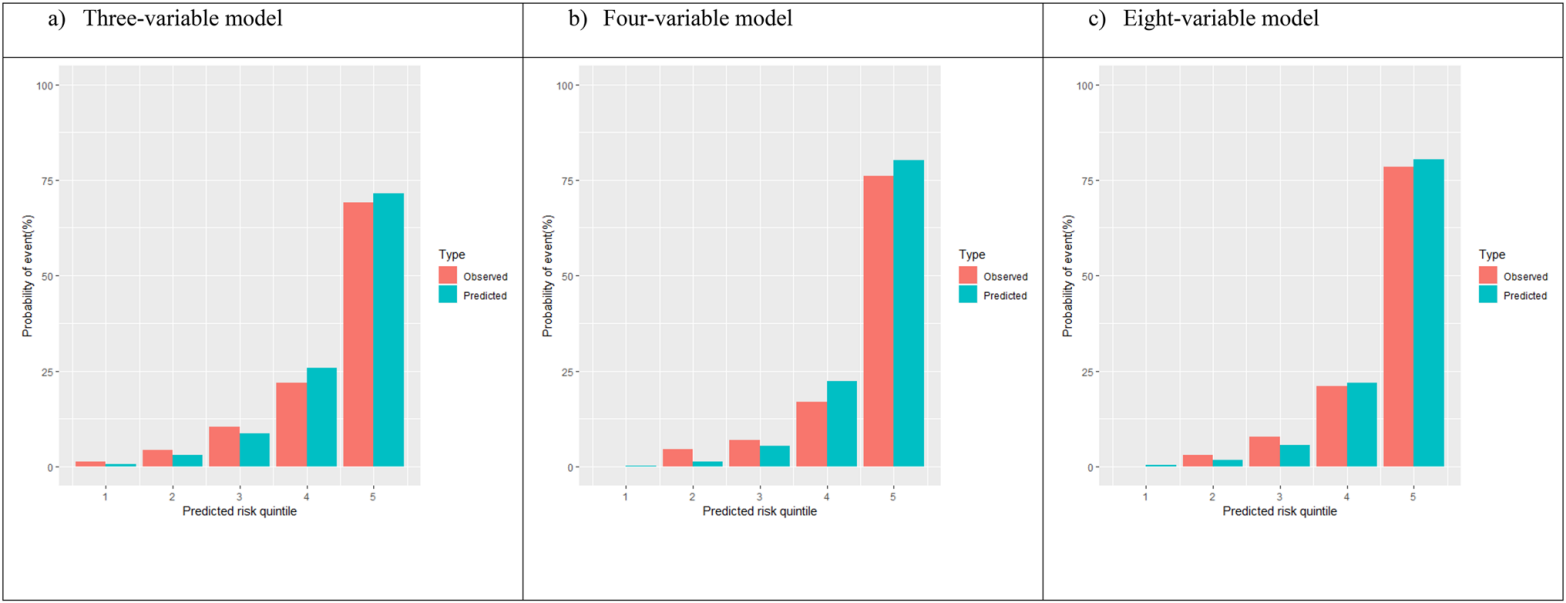
Observed vs predicted probability of kidney failure at 5 years. All three models consistently showed a lower mean observed probability compared to mean predicted probability for the risk quintiles 4 and 5 at 5 years, while the opposite was observed for the risk quintiles 2 and 3 (Fig. 3). The overall mean difference between the observed and predicted risk was 0.51% (*p* = 0.659) for the three-variable model, 0.93% (*p* = 0.602) for the four-variable model, − 0.03% (*p* = 0.967) for the eight-variable model

Of the remaining 406 patients, 71 (17.5%) developed KF, and 112 (27.6%) died during follow-up. Median follow up time was 41 months. Observed vs predicted probability of KF at 5 years was analysed (Fig. 1).

**Fig. 2:**
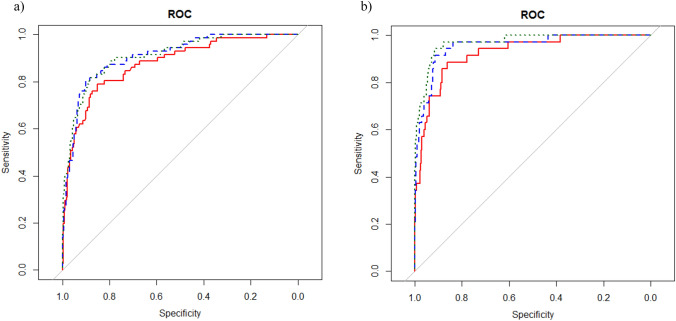
**a** ROC curves at 5 years. Evaluation of the four KFRE models in patients with CKD stages 3–5. ROC-AUCs were 0.888 (95% CI 0.819–0.957), 0.916 (95% CI 0.847–0.985), 0.922 (95% CI 0.853–0.991) for the three-, four-, and eight-variable models, respectively. **b** ROC curves at 2 years. Evaluation of the four KFRE models in patients with CKD stages 3–5. ROC-AUCs were 0.929 (95% CI 0.833–1.0), 0.953 (95% CI 0.857–1.0), 0.952 (95% CI 0.856–1.0) for the three-, four-, and eight-variable models, respectively

Receiver operating characteristic-area under the curve for the three models tested (three-variable, four-variable, eight-variable) appeared similar in predicting both 2 year and 5 year KF risk (Figs. 2a, b). There was some improvement in the ROC-AUCs at 5 years from three-variable to four-variable models (0.888 (95%CI 0.819–0.957) versus 0.916 (95%CI 0.847–0.985)). However, improvement beyond four-variable to eight-variable models showed little change in the ROC-AUC (0.916 (95%CI 0.847–0.985) versus 0.922 (95%CI 0.853–0.991)). These results were similar for ROC-AUCs at 2 years.

After restricting the analysis to patients with CKD stage 3 only and separately for patients with CKD stage 4 only, there was no difference in the ROC-AUCs between three-variable and eight-variable models for CKD stage 3 patients for predicting KF within 5 years. However, the three-variable model seemed to perform poorly for CKD stage 4 patients. There was no difference between the four-variable and eight-variable models (Table 2a). For predicting KF within 2 years, the eight-variable model performed better than both the three- and four-variable models (Table 2b).

**Table 2:** Prediction of kidney failure

	ROC-AUCs (95% CI)	
	CKD stage 3 (*n* = 216)	CKD stage 4 (*n* = 158)
**a** Prediction of kidney failure within 5 years, ROC-AUCs for CKD stages 3 and 4		
Three-variable model	0.76 (0.66, 0.85)	0.73 (0.68, 0.78)
Four-variable model	0.76 (0.67, 0.86)	0.82 (0.78, 0.86)
Eight-variable model	0.76 (0.67, 0.86)	0.82 (0.78, 0.86)

**b** Prediction of kidney failure within 2 years, ROC-AUCs for CKD stages 3 and 4		
Three-variable model	0.878 (0.675, 1.081)	0.748 (0.666, 0.831)
Four-variable model	0.891 (0.719, 1.063)	0.833 (0.784, 0.881)
Eight-variable model	0.995 (− 16.372, 18.362)	0.846 (0.796, 0.897)

The net reclassification index (NRI) further confirmed that there was only a marginal improvement in the performance of the four-variable or eight-variable model over the three-variable model in predicting 5 year and 2 year KF risk (Tables 3a, 3b). The NRI of the four-variable model was 19.5% compared with the three-variable model, while the NRI of the eight-variable model was only 0.5% compared with the four-variable model. Simply, the addition of urinary-ACR to the original three-variable model improved correct classifications considerably. However, the further addition of serum albumin, phosphate, bicarbonate and calcium resulted in only a small increase in net classification accuracy.

**Table 3:** Net reclassification index of kidney failure risk categories

Models	NRI kidney failure % (95% CI)	NRI non-kidney failure % (95% CI)	NRI overall % (95% CI)
**a** Net reclassification index (%), 5 year kidney failure risk categories 0–9.99% (low risk), 10–19.99% (intermediate risk) and ≥ 20% (high risk)			
Four- versus three-variable	7.4 (− 1.2, 14.9)	12.0 (5.3, 14.8)	19.5 (4.8, 22.9)
Eight- versus three-variable	5.7 (0.5, 14.8)	12.2 (8.0, 20.0)	18.0 (12.5, 31.1)
Eight-versus-four-variable	− 0.1 (− 13.1, 15.0)	0.6 (− 5.2, 4.3)	0.5 (− 9.6, 17.8)

**b** Net reclassification index (%), 2 year kidney failure risk categories 0–9.99% (low risk), 10–19.99% (intermediate risk) and ≥ 20% (high risk)			
Four- versus three-variable	3.5 (− 9.2, 22.6)	4.6 (4.9, 5.5)	8.1 (− 3.7, 27.4)
Eight- versus three-variable	− 4.2 (9.9, 21.1)	7.4 (3.4, 9.2)	3.2 (4.4, 27.8)
Eight-versus-four-variable	− 12.2 (− 25.5, 6.7)	2.7 (4.6, 6.4)	− 9.5 (20.9, 7.2)

**Fig. 3:**
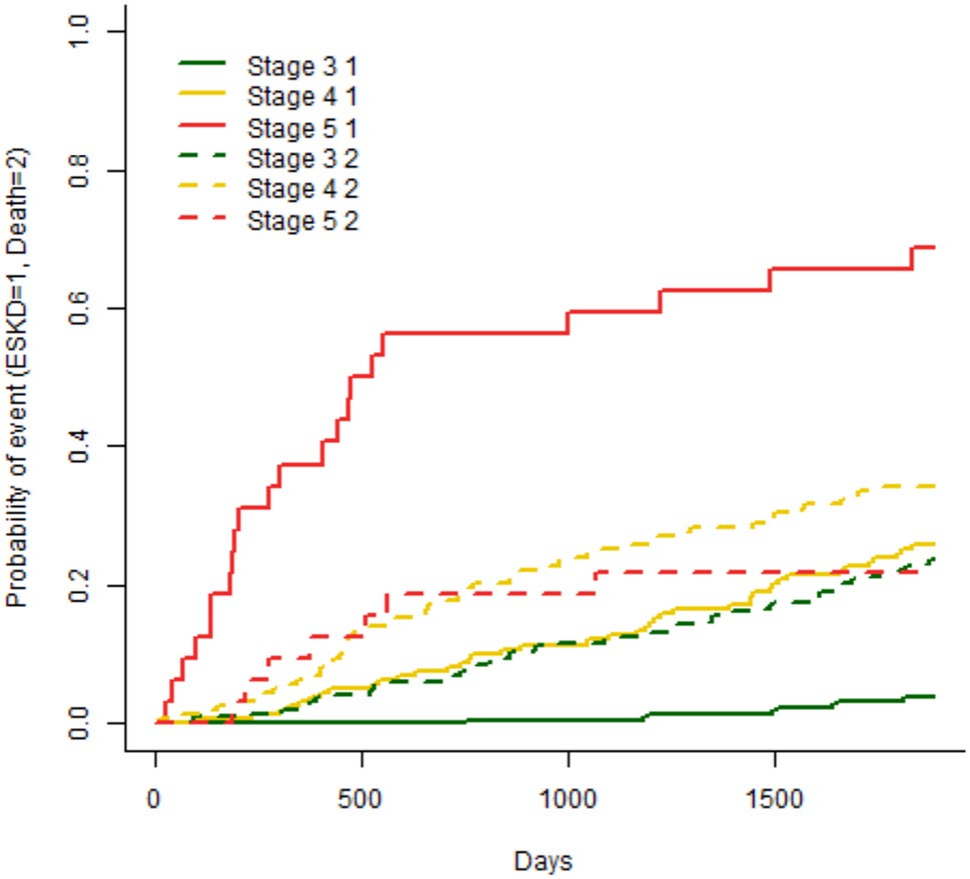
Cause-specific cumulative incidence function (CIF) for progression to KF or death, stratified by CKD stage; with solid lines showing progression to KF and broken lines showing progression to death

A sensitivity analysis of the competing risk analysis model showed that patients with CKD stage 5 progressed to KF faster than those with stage 3 or 4 (Fig. 3). Patients with CKD stage 5 progressed faster to KF than to death, whilst patients with stage 3 or 4 progressed slightly faster to death than to KF.

## Discussion

We demonstrate that KFRE accurately predicted progression to KRT at 2 and 5 years in an Australian metropolitan CKD population. Tangri et al. analysed seven different KFRE models, demonstrating no improvement in model performance with the addition of risk variables obtained from history (diabetes mellitus, hypertension) or clinical examination (blood pressure, weight) [7], and our results concur with this. Of particular note is the rate and unexpected nature of progression related to ethnic diversity. Although 92% of the cohort were of Caucasian background, in those that progressed to KRT, this figure declined to 86%. Sub-group analyses noted that Pacific Islanders progressed more quickly and unexpectedly to KF, perhaps in part due to the burden of diabetes mellitus in this population coupled with documented barriers to accessing health care [12] with knowledge that CKD disproportionately affects Aboriginal, Maori and Pacifica people [13]. This is reported in the First Nations community in Canada, where the rate of progression of kidney disease is higher in this group due to younger age at kidney disease onset, with higher prevalence of diabetes and hypertension [14].

Of interest are those classified by the KFRE as being at low/intermediate risk who do indeed progress to KF, accounting for 13% of those who commenced KRT.

Two thirds of these patients had Autosomal Dominant Polycystic Kidney Disease (ADPKD), indicating a signal for accelerated progression. This variance in progression was also seen by the Canadian group when they compared performance of the equation by disease aetiology [15]. They identified the reason for higher observed than predicted risk to be that disease progression in ADPKD relates more strongly with cyst growth and kidney volume rather than the variables used with the KFRE.

The remaining one third were known to have experienced an incident Acute Kidney Injury (AKI) during the course of the study which changed the original trajectory of their disease.

Unique to this analysis, we were able to undertake cause-specific cumulative incidence function for progression to KF or death, stratified by CKD stage. This demonstrated dramatic differences within the different CKD stages highlighting the interaction of CKD stage, comorbidities and outcomes. We appreciate an amplification effect occurring as a result of CKD stage, and as our study was only over a 5 year period, we predict an ongoing phenomenon in a longer follow-up period.

A strength of our study is that exclusions were limited to those who had ≥one missing variable (*n* = 11) or whose age and/or eGFR fell outside the calibrated range (*n* = 14). Another strength is that we did not censor for mortality as we recognised that the risk of non-renal death was far higher than that of progression to KF. A potential limitation relates to the availability of urinary-ACR in only 14% (*n* = 57) of the cohort, requiring conversion of urine protein:creatinine to ACR [7].

More research is required to evaluate individuals from high-risk populations including Aboriginal, Torres Strait Islander, Maori and Pacific Islander populations as well as patients with ADPKD or AKI to determine specific KFRE accuracy. The KFRE is easy and quick to use with results portrayed in a way that are understandable. In the context of our own and other Australian evidence [10, 11], we aim to incorporate the KFRE into initial referral triage tools to objectively infer risk of the patient referred. With increasing prevalence of kidney disease, the referrals numbers are rising, and this tool will allow appropriate triage from primary care into the hospital outpatient service. This tool can also be used to ascertain which patients can be safely discharged back to their primary care physician, noting higher risks of progression associated with instances such as episodes of AKI. The KFRE can be firmly incorporated into the referral process for access creation and transplant work up, as these steps, if completed correctly, can be the defining moment in a patient’s journey with kidney disease.

## Data Availability

The authors will be happy to consider additional analyses of the anonymised dataset on request. The need for stringent measures to prevent reidentification of individuals within a discrete geographical location and limited time period, however, preclude sharing of patient level datasets in a GDPR compliant form.
